# The effect of spironolactone on calcineurin inhibitor induced nephrotoxicity: a multicenter randomized, double-blind, clinical trial (the SPIREN trial)

**DOI:** 10.1186/s12882-018-0885-6

**Published:** 2018-05-03

**Authors:** Line Aas Mortensen, Helle C. Thiesson, Birgitte Tougaard, Martin Egfjord, Anne Sophie Lind Fischer, Claus Bistrup

**Affiliations:** 1 Department of Nephrology, Odense University Hospital, Sdr. Boulevard 29, DK-5000 Odense C, Denmark; 2 Department of Clinical Research, University of Southern Denmark, Winsløwparken 19, 3. Sal, 5000 Odense C, Denmark; 3 Department of Nephrology, Kolding Hospital, Sygehusvej 24, 6000 Kolding, Denmark; 4 Department of Nephrology, Rigshospitalet, Blegdamsvej 9, 2100 København Ø, Denmark; 5 Department of Nephrology, Aarhus University Hospital, Palle Juul-Jensens Boulevard 99, 8200 Aarhus N, Denmark

**Keywords:** Aldosterone, Mineralocorticoid, Kidney transplantation, Cyclosporine A, Tacrolimus, IFTA, Fibrosis, Glomerular filtration rate

## Abstract

**Background:**

Calcineurin inhibitor induced nephrotoxicity contributes to late allograft failure in kidney transplant patients. Evidence points towards aldosterone to play a role in the development of fibrosis in multiple organs. Animal studies have indicated a beneficial effect of mineralocorticoid receptor antagonists preventing calcineurin inhibitor induced nephrotoxicity. Only few studies have explored this effect in humans. The objective of this study is to evaluate the effect of spironolactone on glomerular filtration rate and fibrosis in kidney transplant patients.

**Method:**

Prospective, double-blind, randomized, clinical trial including 170 prevalent kidney transplant patients. Patients are randomized to spironolactone 25–50 mg/day or placebo for three years. Primary outcome is glomerular filtration rate evaluated by chrome-EDTA clearance. Secondary outcomes are 24-h protein excretion, amount of interstitial fibrosis in renal allograft biopsies, and cardiovascular events. As an exploratory outcome, we aim to identify markers of fibrosis in blood and urine.

**Discussion:**

Long term allograft survival remains a key issue in renal transplantation, partly due to calcineurin inhibitor induced nephrotoxicity. Evidence from animal- and small human studies indicate a beneficial effect of mineralocorticoid receptor antagonism on renal function and fibrosis. This study aims to test this hypothesis in a sufficiently powered randomized clinical trial. Results might influence the future management of long term allograft survival in renal transplantation.

**Trial registration:**

ClinicalTrials.gov identifier (05/17/2012): NCT01602861. EudraCT number (05/31/2011): 2011–002243-98.

## Background

### Background and rationale

The introduction of calcineurin inhibitors (CNI) cyclosporine and tacrolimus as part of immunosuppressive regimens in kidney transplantation has significantly improved short term allograft survival and function [1, 2], but continuous exposure to CNI can result in renal fibrosis ultimately leading to chronic allograft failure. CNI nephrotoxicity is partly caused by vasoconstriction, thereby reducing renal blood flow and inducing oxidative stress, but also CNI directly increase the pro-fibrotic factor transforming growth factor β (TGF-β), apoptosis and macrophage infiltration [3]. Early reports indicated a prevalence of chronic CNI nephrotoxicity of up to 100% after 10 years of cyclosporine treatment [4], although a recent follow up has indicated less nephrotoxicity of modern, tacrolimus-based immunosuppressive protocols [5]. Nevertheless, improving long term allograft function remains a key challenge in renal transplantation.

The mineralocorticoid hormone aldosterone contributes to deleterious pro-fibrotic processes in several organs, including the kidneys [6, 7]. Aldosterone exerts its effects partly via the intracellular mineralocorticoid receptor (MR). Signaling via the MR regulates salt- and water balance, but also prompts inflammation, vasoconstriction and oxidative stress, which ultimately leads to tissue fibrosis [7]. Several large scale studies in congestive heart failure have highlighted the beneficial effect of MR antagonism on survival [8, 9] – partly explained by decreased myocardial fibrosis [10]. In chronic kidney disease, MR antagonism has shown beneficial effects reducing proteinuria, but no study has been sufficiently powered to investigate the effect on kidney function [11]. Evidence from animal models of CNI nephrotoxicity suggests a beneficial effect of MR antagonism on renal blood flow [12, 13], glomerular filtration rate (GFR) [12–18] and renal fibrosis [15–18].

Only a few studies have explored the potential of MR antagonism in kidney transplant recipients. A recent randomized clinical trial included 24 pediatric kidney transplant patients with biopsy-proven chronic allograft nephropathy and did not find a significant effect of eplerenone for 24 months versus placebo regarding GFR, proteinuria and fibrosis. The study was, however, underpowered [19]. One open-label study of 11 kidney transplant patients found significantly reduced levels of proteinuria after 6 months of spironolactone treatment despite unaltered blood pressure [20]. These findings were supported by a retrospective cohort study of 140 proteinuric kidney transplant patients concurrently treated with spironolactone [21]. Additionally, it has been shown that eplerenone given one day before and three days after the transplantation reduces markers of oxidative stress related to ischemia/reperfusion [22]. Hence, current evidence points towards beneficial effects of MR antagonism in kidney transplant patients. This remains to be confirmed in a sufficiently powered prospective trial.

### Objectives and design

The SPIREN trial is an investigator initiated nationwide, multicenter, randomized, double-blind, placebo-controlled clinical trial designed to test the hypothesis that addition of the MR-antagonist spironolactone to standard therapy in prevalent kidney transplant patients will improve long term kidney function and reduce allograft fibrosis.

## Methods

One hundred seventy kidney transplant patients will be included from four Danish hospitals covering all Danish renal transplant centers (Odense, Copenhagen and Aarhus). Patients are recruited from outpatient clinics in the Departments of Nephrology at Odense University Hospital, Kolding Hospital, Rigshospitalet, Copenhagen and Aarhus University Hospital, respectively. Eligibility criteria are listed in Table 1.

**Table 1:** Eligibility criteria

Inclusion criteria	Exclusion criteria
Age > 18 years	Former intolerance of spironolactone
Tacrolimus/cyclosporine treatment	Potassium binder or digoxin treatment
Proteinuria < 3 g/day	Pregnancy or planned pregnancy
Creatinine clearance ≥30 mL/min	Clinically relevant organic, systemic or psychological disorder
Plasma potassium < 5.5 mmol/L	Expectation of non-compliance
Negative pregnancy test at inclusion for women of childbearing potential and adequate contraception throughout the trial	

Wide eligibility criteria have been chosen to obtain a high generalizability of the results. This study includes renal transplant patients with a stable kidney function at the time of inclusion. Even in stable patients, a gradual deterioration of renal function is expected. Previous studies have found an average rate of decline of glomerular filtration rate in of 1–3 mL/min/year [23–26]. Hence, we chose a follow up of 3 years.

**Fig. 1:**
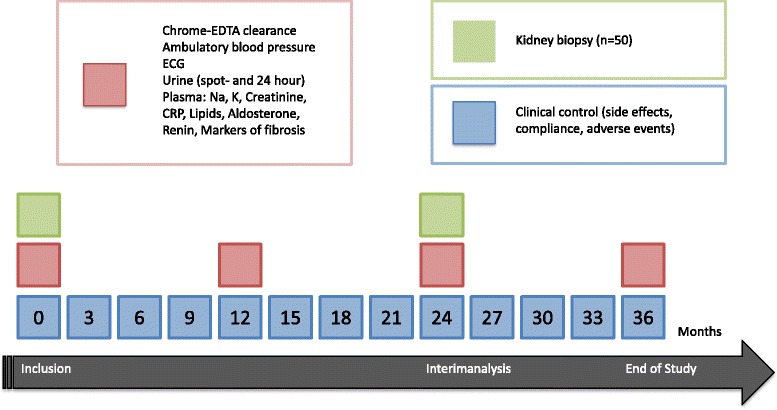
Participant timeline

Patients are randomized 1:1 to spironolactone (Spirix®) or placebo for three years. Initial dosage is 25 mg once daily, which is doubled after three months, if tolerated well. At inclusion and yearly hereafter a standard workup is performed including chrome-EDTA clearance, 24-h urine collection, electrocardiogram, 24-h ambulatory blood pressure measurement and blood- and urine samples (Fig. 1). Anonymized samples are stored at − 80 °C until analyses. In a subgroup (*n* = 50) graft biopsies will be performed at inclusion and after two years. All registrered variables are listed in Table 2.

**Table 2:** All registered variables

Baseline	Every 3 months	Additionally at 0, 1, 2, 3 years		0 and 2 years (*n* = 50)
Date of birth Sex Height Smoking Number of pack years Primary kidney disease Previous dialysis Transplantation date Number of previous transplantations **Comorbidity** Apoplexia Myocardial infarction/cardiac revascularisation Heart failure Hypertension Diabetes Other	**Current medication** **Symptoms** Dizziness Fainting Shortness of breath Gynecomasty Impotence Others **Objective findings** Blood pressure (systolic) Blood pressure (diastolic) Pulse Bodyweight **Blood samples** Sodium Potassium Creatinine **Adverse events** **Compliance to study drug**	**Objective findings** Heart stethoscopy Lung stethoscopy Edema ECG Chrome-EDTA clearance **Blood samples** CRP Total cholesterol HDL LDL Triglyceride Markers of fibrosis **24 h urine sample** Creatinine clearance Proteinuria U-sodium U-potassium Markers of fibrosis	**Ambulatory blood pressure** SBP DBP SBP-day SPB-night DBP-day DBP-night Average pulse	**Kidney biopsy** Amount of fibrosis (%) Banff-score

*ECG* Electrocardiogram, *CRP* C-reactive protein, *HDL* High density lipoprotein, *LDL* Low density lipoprotein, *SBP* Systolic blood pressure, *DBP* Diastolic blood pressure

Side effects, adverse events and compliance to the study drug are evaluated at project visits every three months. Compliance is evaluated by counting tablets.

### Outcome

Outcomes are summarized in Table 3.

**Table 3:** Study outcomes

Primary endpoint	Secondary endpoints
Chrome-EDTA clearance	24 h urinary protein excretion
	Renal fibrosis (morphology)
	Cardiovascular events
	Biomarkers of fibrosis in tissue, blood and urine

The primary endpoint is renal function evaluated by chrome-EDTA clearance, in which glomerular filtration rate is calculated from the area under the plasma clearance curve after a single intravenous doses of ^51^CrEDTA. Plasma concentrations of ^51^CrEDTA can be determined with a single blood sample by measuring residual radioactivity 4 h after the injection or by multiple samples 4, 5 and 6 h after the injection. For centers using a single blood sample after 4 h, an additional blood sample is performed 24 h after the injection in male patients with plasma creatinine ≥200 μmol/L and female patients with plasma creatinine ≥150 μmol/L. In repeat chrome-EDTA clearance measurements the coefficient of variation is 8–10% [27].

Secondary endpoints include 24-h urinary protein excretion, cardiovascular events (death, myocardial infarction, stroke or peripheral arterial thrombosis) and renal fibrosis. For the latter, Masson Trichrome stained sections of kidney allograft biopsies will be used for measuring Banff chronicity scores and calculating morphologic fibrosis by point counting. Point counting provides a quantitative and reproducible estimation of the extent of fibrosis [28]. In brief, the software systematically selects sections of renal cortex and superimposes a grid on a computerized image. The extent of fibrosis is determined by counting the fraction of intersection points that overlie fibrotic areas relative to normal tissue. Limitations of both Banff scoring and point counting are the risk of sampling error as well as the somewhat subjective evaluation of fibrosis. In this study all biopsies will be reviewed by the same pathologist to avoid inter-observer variability.

As an exploratory outcome we aim to identify possible biomarkers of fibrosis in blood and urine. Kidney fibrosis is currently diagnosed by renal biopsy. Besides being time-consuming, it is an invasive procedure and as such, it implies a risk of complications including bleeding, pain and infection. Hence, much effort has gone into identifying non-invasive markers of fibrosis in blood or urine. The fibrotic process is a cascade of factors including inflammation, tissue hypoxia and various pro-fibrotic cytokines and growth factors [29]. Animal studies of MR antagonism in CNI nephrotoxicity have identified several markers involved in the fibrogenic process that were regulated by MR antagonists [30]. Simultaneous plasma−/urine samples and renal biopsies allow for the possible identification of non-invasive markers of fibrosis. Further, studies of markers in blood and urine might contribute to our understanding of the molecular mechanisms of MR antagonism. Specific markers of interest will be defined when all samples have been obtained.

### Safety

Plasma potassium and -creatinine levels are monitored closely at initiation of therapy and after any dose adjustment. Sustained potassium levels > 5.8 mmol/L will result in reduction of dosage to 25 mg per day. In case of hyperkalemia on 25 mg per day, the patient will be withdrawn from the study. Potassium binding resins will only be used to handle cases of acute hyperkalemia, thus chronic treatment with resins is not allowed. Hyperkalemia is registered as an adverse event in case of an absolute increase of > 1.5 mmol/L or any increase to a level above 5.5 mmol/L. All significant increases in plasma creatinine are evaluated by study investigators, and in case of doubt of the etiology, the study drug is discontinued until renal function has stabilized. All adverse events registered throughout participation in the study will be recorded. Serious adverse events are defined according to Good Clinical Practice as events that result in death, are life threatening, require hospitalization, cause prolongation of existing hospitalization or result in persistent or significant disability/incapacity. The project safety board continuously monitors serious adverse events to identify general study safety issues.

### Withdrawal

Withdrawal criteria include a sustained compliance of less than 80%, hyperkalemia despite reduction of dosage or intolerable side effects.

### Sample size

Assuming a standard deviation in chrome-EDTA clearance of 10 mL/min, a sample size of 126 patients (63 in each group) will be able to detect a difference in chrome-EDTA clearance of 5 mL/min between the groups with 80% power and a significance level of 0.05. With an expected dropout rate of 15%, the planned total was set to 170 patients.

### Allocation and blinding

After completing the baseline workup (Fig. 1), patients are allocated to consecutive randomization numbers. Randomization has been performed by the pharmacy at Odense University Hospital in blocks of four. The allocation code is blinded to both patients and investigators and is solely known by the pharmacy. Opaque envelopes containing the individual allocation are available on site in case of the need for emergency unblinding. If the allocation code is revealed before completing the trial the patient will be excluded.

### Data management

All data will be documented for each visit in the respective Case Report Form and subsequently entered into the SPIREN project database. Data will be secured by double entry. The project database has been developed in cooperation with the Database Unit of the Region of Southern Denmark and complies with the standards of Good Clinical Practice.

### Statistical methods

All statistical analyses will be done using STATA software. All generated variables will be documented by do-files. The main analysis will be performed as intention-to-treat using last-observation-carried-forward. Sub-analyses will include per protocol analyses and “best case”/“worst case” analyses. Where appropriate, variables will be analyzed using parametric or non-parametric tests.

### Trial status

The first patient was included in January 2013. An interim analysis was performed in September 2015 including 40 patients after two years of participation [31]. The interim analysis evaluated the rates of decline in kidney function between the two groups to ensure the safety of the study drug and found no difference between the two groups. In November 2017 a total of 142 patients were active in the study and 41 baseline biopsies had been performed. Inclusion is expected to be complete by 2018.

## Discussion

Long term kidney graft survival is a key issue in transplant medicine. Chronic CNI nephrotoxicity contributes to late allograft loss [3]. There is increasing evidence, that aldosterone plays a role in detrimental processes beyond fluid and sodium homeostasis e.g. inflammation and fibrosis, thereby possibly contributing to allograft failure [7]. Several animal studies have shown an effect of MR antagonism in reducing CNI nephrotoxicity [30]. The few available human studies addressing this hypothesis have been underpowered or focused on short term effects [19, 22]. This study aims to provide sufficiently powered evidence regarding the effect of long term MR antagonism on GFR and the development of kidney fibrosis in prevalent kidney transplant patients.

Concerns have been raised regarding MR antagonism in patients with impaired renal function due to the risk of hyperkalemia, which is a potentially lethal side-effect. In the wake of the Randomized Aldactone Evaluation Study (RALES) - a randomized, clinical trial investigating the effect of spironolactone in patients with congestive heart failure and serum creatinine below 221 μmol/L - there was a significant increase in hospitalizations due to hyperkalemia from 2.4 to 11.0 per 1000 patients [32]. The risk of hyperkalemia increases as GFR deteriorates. One safety study of MR antagonist eplerenone 25 mg/day for 8 weeks in 31 kidney transplant patients found a significant increase in plasma potassium during eplerenone treatment, however there was only one incidence of moderate hyperkalemia (> 5.5 mmol/L) and no patients were withdrawn from the study due to hyperkalemia. It was concluded, that MR antagonism is safe in kidney transplant patients with a GFR > 30 mL/min, but warrants close monitoring of plasma-potassium [33].

Another concern regarding MR antagonism is the observation that plasma creatinine levels tend to increase at the initiation of therapy. This effect has also been observed in studies of MR antagonism in chronic kidney disease [34]. In RALES, spironolactone significantly increased the occurrence of worsening renal function - defined as a reduction of eGFR> 30% - however, this did not impact the relative mortality risk in this group. Interestingly, worsening renal function was associated with an increased adjusted mortality risk in the placebo group, but not in the spironolactone group [35]. Although the increase in plasma creatinine is reversible when MR antagonists are discontinued, worsening renal function poses a diagnostic challenge with several possible etiologies in the kidney transplant population (e.g. acute rejection, post-renal obstruction, BK-virus nephropathy etc.). If in doubt, MR antagonists should be discontinued and appropriate diagnostic measures performed.

Despite potential pitfalls using MR antagonists in kidney transplant patients, evidence from animal and small human studies indicate a potential for improving long term allograft function. This study is, to our knowledge, the first to investigate the effect of MR antagonism on long term allograft function in adult kidney transplant patients in a prospective, randomized fashion. Results will contribute to current evidence and possibly influence the future management of kidney transplant patients with regards to improving long term allograft survival.

## Abbreviations

CNI: Calcineurin inhibitors
GFR: Glomerular filtration rate
MR: Mineralocorticoid receptor
RALES: The Randomized Aldactone Evaluation Study
TGF-β: Transforming growth factor β
